# Ethical challenges and regulatory pathways for artificial intelligence in rheumatology

**DOI:** 10.1093/rap/rkaf035

**Published:** 2025-04-18

**Authors:** Vincenzo Venerito, Latika Gupta, Saverio Mileto, Florenzo Iannone, Emre Bilgin

**Affiliations:** Rheumatology Unit, Department of Precision and Regenerative Medicine-Ionian Area, University of Bari “Aldo Moro”, Bari, Italy; Department of Rheumatology, Royal Wolverhampton Hospitals NHS Trust, Wolverhampton, UK; School of Infection, Inflammation and Immunology, College of Medicine and Health, University of Birmingham, Birmingham, UK; Key Law, Bari, Italy; Rheumatology Unit, Department of Precision and Regenerative Medicine-Ionian Area, University of Bari “Aldo Moro”, Bari, Italy; Division of Rheumatology, Department of Internal Medicine, Sakarya University Faculty of Medicine, Sakarya, Turkey

Key message

- AI integration in rheumatology is facing unique ethical and regulatory challenges due to longitudinal patient data complexity.

Dear Editor, The integration of artificial intelligence (AI) into rheumatology practice raises important ethical considerations that warrant careful attention [1–3]. The chronic and complex nature of rheumatologic conditions presents unique challenges for AI implementation that deserve focused discussion.

Rheumatology stands apart from other medical specialties due to the longitudinal accumulation of patient data over years or decades of disease management. Patients with conditions like rheumatoid arthritis, systemic lupus erythematosus, and other autoimmune diseases generate vast amounts of data through regular monitoring of disease activity, medication responses and periodic flares. This longitudinal data presents both opportunities and ethical challenges for AI applications.

The European Union (EU) AI Act [4] stands as the first comprehensive legislation specifically addressing AI systems in healthcare and other domains. While other jurisdictions rely on policy frameworks and guidelines, this legislation establishes binding requirements for AI development and deployment. This distinction is particularly relevant for rheumatology, where AI applications could involve complex decision support systems managing sensitive patient data over extended periods.

Disease flare prediction exemplifies the complex interplay between AI capabilities and regulatory requirements in rheumatology. For instance, under the EU AI Act, such predictive systems would be classified as ‘high-risk’ AI since they influence medical decisions and patient outcomes [5, 6]. This classification brings significant regulatory obligations, including requirements for robust risk management systems, high-quality training data and detailed technical documentation. For flare prediction specifically, these requirements are crucial given the serious consequences of prediction errors. While early prediction could revolutionize pre-emptive treatment, a false positive prediction might lead to unnecessary treatment intensification with potential adverse effects, while a missed prediction could result in preventable organ damage. The high-risk designation means these systems must maintain rigorous logging capabilities and enable human oversight of predictions, allowing rheumatologists to understand and potentially override AI recommendations when clinically appropriate.

The regulatory landscape for medical AI varies significantly across jurisdictions. The EU AI Act establishes a risk-based regulatory approach with binding legal requirements. In contrast, the US Food and Drug Administration implements a product-based classification system through regulatory guidelines, focusing on safety and effectiveness verification for AI-driven medical devices.

The UK has adopted a distinctive sector-specific approach, combining targeted oversight through existing regulatory bodies like the Medicines and Healthcare products Regulatory Agency with new coordination mechanisms through the Digital Regulation Cooperation Forum. This is supported by the AI Safety Institute and upcoming AI Bill, maintaining flexibility while implementing mandatory requirements for high-risk systems.

The implications of AI regulation for rheumatology extend beyond flare prediction. Any AI system used for medical diagnosis, patient triage or treatment planning falls under the high-risk category and must meet stringent requirements for accuracy, robustness and cybersecurity (Table 1). Healthcare providers deploying these systems must implement quality management systems, ensure ongoing monitoring and maintain detailed documentation of the AI system’s development and validation.

**Table 1.** Ethical and safety considerations for AI systems in rheumatology under the EU AI Act.

Challenge	Potential risks	Ethical aspects (EU AI Act)	Safety aspects (EU AI Act)
Bias in training data	Discriminatory outcomes due to underrepresented populations	Ensure diversity and inclusivity in training datasets	Robust dataset quality checks and representation
Prediction errors (false positives/negatives)	False alarms or missed disease flares impacting patient care	Mitigate harm by minimizing prediction errors	Rigorous testing to avoid critical prediction errors
Data privacy and security	Breach of sensitive personal and genetic health data	Adhere to the General Data Protection Regulation and protect patient confidentiality	Edge computing to ensure local data processing and security
Transparency and explainability	Black-box algorithms leading to lack of trust and usability	Provide clear and understandable AI system outputs	Transparency in model decision-making processes
Human oversight	Overreliance on AI without sufficient human intervention	Enable effective human control and accountability	Implement mechanisms for clinician overrides
Compliance with EU AI Act requirements	Non-compliance with mandatory high-risk system regulations	Follow strict documentation, validation and risk management	Regular audits and adherence to regulatory standards
Lifecycle monitoring and documentation	Decreased system performance or safety post-deployment	Maintain transparency and accountability throughout the AI lifecycle	Post-deployment monitoring and periodic revalidation of AI systems

Data security takes on heightened importance in rheumatology given the sensitive nature of genetic information and autoimmune histories. The EU AI Act mandates specific data governance practices for high-risk AI systems, including requirements for training data quality and privacy protection [1]. For rheumatology, where the chronic nature of diseases means breaches could expose decades of personal health information, these requirements are particularly relevant. One promising approach to address these concerns is the implementation of locally run algorithms within hospital systems. Edge computing and local large language models that operate entirely within a healthcare institution’s infrastructure can process sensitive patient data without exposing it to external servers, potentially offering a path to compliance with both the AI Act’s requirements and data protection regulations [2].

A crucial element that requires further attention is health equity. AI applications in rheumatology risk exacerbating existing disparities in disease detection, treatment access and patient outcomes if training data are not adequately representative. Many rheumatic diseases present differently across ethnic groups, yet AI training datasets may not reflect this diversity. Without robust safeguards, AI-driven tools risk reinforcing systemic biases rather than mitigating them. Addressing this concern requires greater transparency in dataset composition, regulatory mandates for inclusivity and ongoing performance monitoring to assess AI-driven disparities in healthcare outcomes [8].

The doctor–patient relationship in rheumatology, built over years of managing chronic disease, must not be undermined by AI implementation. Patient perspectives remain central to successful AI integration. Early engagement through patient panels in AI development can help identify concerns and priorities. Surveys indicate that while patients welcome AI’s potential to enhance care, they worry about privacy and the ‘dehumanization’ of medical care. The EU AI Act’s emphasis on human oversight aligns with this concern, requiring that high-risk AI systems be designed to be effectively overseen by humans [6]. While AI can process vast amounts of longitudinal data, the rheumatologist’s accumulated knowledge of individual patient patterns and responses remains invaluable. Clear frameworks for integrating AI insights with clinical expertise are essential and must satisfy both regulatory requirements and clinical needs.

AI developers face complex compliance requirements, including regular audits, transparency in algorithm development and robust documentation of validation processes. This extends to ongoing monitoring and updates of AI systems post-deployment. The question of liability remains critical—when AI contributes to clinical decisions, clear frameworks are needed to delineate responsibility between developers, healthcare providers and clinicians. Insurance policies must evolve to cover AI-specific risks while maintaining affordability of implementation.

Looking ahead, the emergence of the EU AI Act as the first comprehensive AI legislation creates an opportunity for global harmonization of AI governance in healthcare. International collaborations between regulatory agencies, professional societies (such as EULAR and ACR) and AI developers could facilitate the development of unified ethical frameworks. The World Health Organization continues to develop guidelines and technical standards that bridge different regulatory approaches, potentially facilitating greater international alignment [7].

This means developing systems that can process complex longitudinal data while maintaining privacy, predict disease trajectories while allowing for clinical judgment and enhance rather than replace the doctor–patient relationship. The unique challenges of rheumatology—from the variability of disease manifestations to the complexity of treatment decisions—make it an important testing ground for responsible AI implementation in medicine.

## Data Availability

Data are available upon reasonable request by any qualified researchers who engage in rigorous, independent scientific research and will be provided following review and approval of a research proposal and Statistical Analysis Plan (SAP) and execution of a Data Sharing Agreement (DSA). All data relevant to the study are included in the article.
